# Association between the Use of Health Services, Cardiovascular Risk Factors and Metabolic Syndrome in Mexican Adults

**DOI:** 10.3390/ijerph18105336

**Published:** 2021-05-17

**Authors:** María Araceli Ortiz-Rodríguez, María Vanessa Aldaz-Rodríguez, Luz María González-Robledo, Antonio Villa, Cristina Bouzas, Rosario Pastor, Josep A. Tur

**Affiliations:** 1 Faculty of Nutrition, Autonomous University of the State of Morelos, Cuernavaca 62350, Mexico; araceli.ortiz@uaem.mx (M.A.O.-R.); luz.gonzalez@uaem.mx (L.M.G.-R.); 2 Faculty of Medicine, National Autonomous University of Mexico, México City 04360, Mexico; vanessa.aldaz@gmail.com (M.V.A.-R.); arvillamx@yahoo.com (A.V.); 3 Research Group on Community Nutrition and Oxidative Stress, University of the Balearic Islands-IUNICS & IDISBA, 07122 Palma de Mallorca, Spain; cristina.bouzas@uib.es (C.B.); rosario.pastor@ucavila.es (R.P.); 4 CIBER Physiopathology of Obesity and Nutrition (CIBEROBN), Institute of Health Carlos III (ISCIII), 28029 Madrid, Spain; 5 Faculty of Health Sciences, Catholic University of Avila, 05005 Avila, Spain

**Keywords:** health services, metabolic syndrome, cardiovascular risk factors, Mexico

## Abstract

*Background*: The use of health services is a complex behavioral phenomenon affected by multiple factors (availability, distance, cost, quality, attitudes, cultural beliefs, socioeconomic characteristics, and individuals’ self-perception of health). Mexico has a segmented health system, and the access to it depends on the labor insertion and the population’s ability to pay. *Objective*: To assess association between use of health services and cardiovascular and metabolic syndrome risk factors among Mexican adults. *Methods*: Analytical cross-sectional nationally representative study carried out on Mexican adults (≥20-year-old adults of both sexes; n = 4595). Socioeconomic factors, geographic area, health care coverage, information about the use of health services, previous medical diagnoses of diabetes and hypertension, and smoking were assessed. Anthropometrics, triglyceride, total cholesterol, HDL-cholesterol, and glucose plasma levels were measured. Metabolic syndrome (MetS) and cardiovascular risk factors were assessed. Prevalences were expressed in terms of percentages, and significant differences were calculated using χ^2^ test. Univariate and multivariate analysis was performed to evaluate the association between the use of health services and cardiovascular risk factors and sociodemographic variables. *Results*: The probability of using health services is higher and more significant in subjects with obesity, diabetes (OR (95% CI): 1.73 (1.49–2.00; *p* < 0.001), hypertension (OR (95% CI): 1.29 (1.14–1.45; *p* < 0.001), hypertriglyceridemia (OR (95% CI): 1.30 (1.15–1.46; *p* < 0.001), and in those with hypercholesterolemia (OR (95% CI): 1.23 (1.03–1.39; *p* = 0.001). *Conclusions*: Among health service users, there is a positive significant association between the use of health services and the presence of metabolic syndrome, obesity, diabetes, hypertension, hypertriglyceridemia, and hypercholesterolemia.

## 1. Introduction

The use of health services is a complex behavioral phenomenon that is affected by multiple factors including availability, distance, cost, and quality of services, as well as attitudes, cultural beliefs, socioeconomic characteristics, and individuals’ self-perception of health [1,2,3]. In recent decades, there has been an increase in the demand and use of health services due to cardiovascular risk factors and metabolic syndrome, particularly in low- and middle-income countries, due to the physical and progressive deterioration, disability, and high comorbidities and mortality [4,5]. In Mexico, the main causes of mortality are heart diseases (20.1%), diabetes (15.2%), malignant tumors (12%) and liver diseases (5.5%) [6]. Obesity, diabetes mellitus and arterial hypertension occupy the eighth, tenth and eleventh places as main causes of general morbidity [7], which are conditions closely related to the metabolic syndrome (MetS), generating great demands for health services at several levels of care.

Mexico has a segmented health system which coexists with social security institutions (Institute of Social Security, IMSS; Institute of Security and Social Services of State Workers, ISSSTE; Health Services for Armed Forces, SEDENA and SEMAR; Health Services for workers of Mexican Petroleum, PEMEX; and others); institutions from the public system (Popular Security, which was substituted in 2020 by the Health Institute for Well-being, INSABI), as well as institutions from the private sector [8]. Access to these institutions and services depend on the labor insertion and the population’s ability to pay, except for INSABI which is health assistance free of charge [9].

The aim of this study was to assess associations between the use of health services and cardiovascular and metabolic syndrome risk factors among Mexican adults.

## 2. Methods

### 2.1. Design

An analytical cross-sectional nationally representative study was carried out on workers and pensioners affiliated with ISSSTE in Mexico (2007), which covers health services of 8.8% of Mexicans [8].

### 2.2. Subjects

The population was selected by means of a multiple-step, random selection, considering first the location (35 ISSSTE delegations), delegation size (two strata: <3000 and ≥3000 workers and relatives per delegation), and sex of participants, and then participants were randomization into subgroups, with delegations being the primary sampling units, and individuals within these delegations comprising the final sample units. Quality of cluster was 0.8; ratio of sizes: 1.22. The theoretical sample size was calculated from 3500 workers and 1085 pensioners to provide a confidence level = 95%, expected proportion = 50%, accuracy = 2.027 and design effect = 1.5. Subjects were randomly selected from the ISSSTE archives, invited by letter to make an appointment for the interview, and afterwards confirmed by phone call. When the chosen subject did not answer after three telephone calls, declined to participate, or there were involuntary non-participations due to census error, another subject was randomly chosen from the general file. A total of 80 subjects did not participate for the reasons described above, and then a second selection was done. The final sample size was 4595 individuals (≥20-year-old adults of both sexes).

The study was conducted according to the guidelines laid down in the Declaration of Helsinki, and the study protocol was approved by the Committee of Ethics in Research of the National Institute of Public Health, Cuernavaca, Morelos, Mexico (ref. 613-CI-210-2007). All participants were informed of the purpose and methods of this study and signed the informed consent prior to enrolment.

### 2.3. General Questionnaire

A questionnaire [10] incorporating the following questions was used: gender, age (20–39 y.o., 40–59 y.o., and ≥60 y.o.), region (north: Baja California, Baja California Sur, Chihuahua, Coahuila, Nuevo León, Sinaloa, Sonora, and Tamaulipas; central-west: Federal District, State of Mexico, Hidalgo, Morelos, Puebla, Querétaro, and Tlaxcala; central: Aguascalientes, Colima, Durango, Guanajuato, Jalisco, Michoacán, Nayarit, San Luis Potosí, and Zacatecas; and south-south east: Campeche, Chiapas, Guerrero, Oaxaca, Quintana Roo, Tabasco, Veracruz, and Yucatán), scholar level (primary school, high school, preparation for the university and university), frequency of use of health services (always, usually, sometimes, rarely, and never), previous medical diagnoses of diabetes and hypertension. Smoking was also assessed, which was self-reported and categorized as “current” (subjects who had smoked at least 100 cigarettes during their life and were currently smokers) and “ex-smokers” (those who had smoked at least 100 cigarettes during their lifetime, but they were not smokers currently).

### 2.4. Anthropometric Measurements

Weight, height, and waist circumference measurements were obtained by trained personnel. Height was measured using a mobile stadiometer (Seca 213, Hamburg, Germany), with an accuracy of 0.5 cm, with the subject’s head in the Frankfurt plane. Body weight was determined to the nearest 100 g using a digital scale (Seca 354, Hamburg, Germany), with the subjects in an upright position, barefoot, fasting and wearing light clothing, which was counted by subtracting 300 g from the average weight. Height and weight were measured in duplicate, and the average of each variable was used for calculations and analysis. Body mass index (BMI) was calculated as body weight (kg) divided by height squared (m). The WHO categories were used for normal weight (BMI = 18.5 to ≤25 kg/m^2^), overweight (BMI = 25 to ≤30 kg/m^2^) and obesity (≥30 kg/m^2^). Waist circumference (CCi) was measured with a fiberglass tape measure (Seca 120, Hamburg, Germany), with subjects asked to stand on a flat surface in a relaxed position, with their feet together. CCi was measured as the smallest horizontal circumference between the costal margins and the iliac crests at minimum respiration. Measurements were made with a precision of 0.1 cm. Two CCi measurements were made, and the mean of the two readings was taken as the final value.

### 2.5. Biochemical Analyses

Blood samples were collected in tubes containing ethylene diamine tetra acetic acid (EDTA). Plasma was separated by centrifugation (3000 rpm; 4 °C; 20 min). Triglyceride, total cholesterol, HDL-cholesterol, and glucose levels were measured by automated enzymatic methods (Beckman Synchron CX, Brea, CA, USA). LDL-cholesterol was calculated by the Friedewald formula [11]. Insulin levels were determined using microparticle enzyme immunoassay (MEIA) (Abbot’s Axym System, Green Oaks, IL, USA) and apo B levels by kinetic nephelometry (Beckman Immage, Brea, CA, USA). Aliquots of plasma were stored at −70 °C. The laboratory was certified by the American College of Clinical Chemistry.

### 2.6. Metabolic Syndrome

Metabolic syndrome (MetS) was defined according to the Joint Interim Statement of the International Diabetes Federation Task Force on Epidemiology and Prevention; National Heart, Lung, and Blood Institute; American Heart Association; World Heart Federation; International Atherosclerosis Society; and International Association for the Study of Obesity [12], including three or more of the following criteria: waist circumference ≥94 cm in men and ≥80 cm in women (for Europids) or ≥90 cm in men and ≥80 cm in women (for ethnic Central and South America), elevated triglyceride levels ≥ 150 mg/dL, receiving medical treatment for elevated triglyceride (TG), HDL-cholesterol lower than <40 mg/dL in men and <50 mg/dl in women, elevated SBP ≥ 130 mm Hg or elevated DBP ≥ 85 mm Hg or having a medical diagnosis of hypertension, and an elevated fasting glucose ≥ 100 mg/dL or a medical diagnosis of diabetes mellitus.

### 2.7. Cardiovascular Risk Factors

Several cardiovascular risk factors were analyzed, which may be non-modifiable (age and sex) or modifiable, such as hypertension, smoking, hypercholesterolemia, diabetes mellitus, overweight/obesity, hypertriglyceridemia, low HDL-cholesterol level, and total cholesterol [13].

### 2.8. Statistics

Analyses were performed with the SPSS statistical software package version 25.0 (SPSS Inc., Chicago, IL, USA). Prevalence was expressed in terms of percentages. Significant differences in prevalence were calculated using χ^2^ test. To evaluate the association between the use of health services (dependent variable) and the cardiovascular risk factors and sociodemographic variables (independent variables), logistic regression analyses with the estimation of the corresponding odds ratios (unadjusted and adjusted ORs) and the 95% confidence interval (CI) were used. Multivariate analyses (multiple logistic regressions considering the simultaneous effect of each explanatory variables) were performed. The level of significance was considered at *p* < 0.05 for all statistics.

## 3. Results

### 3.1. Use of Health Services According to Sociodemographic Characteristics

Women used more health services than men (71.5% and 28.5%, respectively; *p* < 0.001). The use of health services was higher in subjects from 40 to 59 years (50.9%; *p* < 0.001) and in those who studied at university (40.9%; *p* < 0.001) (Table 1).

### 3.2. Use of Health Services According to Prevalence of Metabolic Syndrome and Its Components

Around half (51.3%) of those who used health services showed MetS. Those with higher triglyceride levels (55.5%), low HDL-cholesterol levels (62.7%), abdominal obesity (50.5%), and hypertension (50.6%), and also those who had normal fasting glycemia, showed greater use of health services (Table 2).

### 3.3. Use of Health Services According to Cardiovascular Risk Factors

Among people with cardiovascular risk factors, those who most used health services were active smokers (65.5%), or those with overweight/obesity (42.8% and 33.2%, respectively), hypertriglyceridemia (60.1%), high cholesterol (65.1%), or low HDL-cholesterol level (62.7%). In contrast, people without diabetes, hypertension and/or MetS showed a scarce use of health services (Table 3).

### 3.4. Association of the Use of Health Services with Sociodemographic Characteristics, Cardiovascular Risk Factors and Metabolic Syndrome

Table 4 shows association of the use of health services with sociodemographic characteristics, cardiovascular risk factors and metabolic syndrome (unadjusted OR).

The study of the association of the use of health services with sociodemographic variables shows that there is a higher and significant probability of the use of health services in women (OR (95% CI): 1.56 (1. 37–1.76; *p* < 0.001), subjects 60 years of age or older, those with a lower level of education than primary school and those living in the south-south east region.

Regarding the association of the use of health services with cardiovascular risk factors, it was observed that the probability of use of health services is higher and significant in subjects with obesity (OR (95% CI): 1 (ref.)]), with diabetes (OR (95% CI): 1.73 (1.49–2.00; *p* < 0.001), hypertension (OR (95% CI): 1.29 (1.14–1.45; *p* < 0.001), hypertriglyceridemia (OR (95% CI): 1.30 (1.15–1.46; *p* < 0.001), and those with hypercholesterolemia (OR (95% CI): 1.23 (1.03–1.39; *p* = 0.001).

Finally, it was observed that subjects with MetS have a higher and more significant probability of using health services (OR (95% CI): 1.48 (1.31–1.67; *p* < 0.001).

When a step-by-step model was applied for the calculation of logistic regression (adjusted OR), the variables that turned out to have a greater association to the use of health services were sex (women (OR [95% CI]: 1.77 (1.54–2.03; *p* < 0.001)), age (60 or more years (OR [95% CI]: 1 (ref.)), school level (lower primary school (OR (95% CI): 4.27 (0.90–20.24.; *p* = 0.050)), region (south-south east (OR (95% CI): 1 (ref.)), diabetes (presence of diabetes (OR [95% C]: 1.37 (1.16–1.63; *p* < 0.001)) and hypertriglyceridemia (presence of hypertriglyceridemia (OR (95% CI): 1.25 (1.09–1.43; *p* < 0.001)) (Table 5).

## 4. Discussion

The probability analysis of the current study showed a significant positive association between the use of health services and the presence of metabolic syndrome and cardiovascular risk factors (obesity, diabetes, hypertension, hypertriglyceridemia, and hypercholesterolemia). It should be noted that the variables that showed greater association with the use of health services were diabetes and hypertriglyceridemia. These results are like those observed in previous studies [4,13,14,15]. The greater use of the doctor’s office by chronic patients could be explained by the exacerbation of chronic pathologies or new acute processes which demands medical attention, as well as scheduled check-ups [4]. It is also important to highlight that people with chronic diseases tend to show progressive deterioration, gradual loss of autonomy, poorer quality of life, high morbidity, and mortality, and therefore they use health services more [4,13,14,15].

More than 50% of the eligible population did not use the available care services. These results are like previous studies published in the scientific literature, confirming that the greater the presence of chronic diseases or risk factors, the greater the use of health services [16,17,18].

It is important to note that these diseases are chronic, progressive, and complicated from a clinical point of view. Furthermore, they can be disabling or leave residual sequelae, especially in the absence of effective control programs. The paradox is that many of these diseases are preventable, but when they occur, they demand prolonged, continuous, expensive, and overly complex care [19].

Several studies pointed out that use of health services is not an indicator of access to health services, and there is a proportion of people, ranging between 60% and 70%, who require it and do not have access to it. Such a phenomenon has been described since the 1960s as the “iceberg of disease” [19]. There may also be a pattern of overuse, unjustified from a medical point of view, related to the clinical condition, frequency, intensity, or sophistication of the service that does not compensate for the benefit. This overuse is based on multiple causes [20,21,22], such as self-perception of loss of health or well-being, symptoms, type of disease, time of disability, as well as the patient’s perception of biological or social restriction to develop daily activities [20,23,24]. The wish for care also depends on beliefs on health, tolerance of discomfort, and trust in the health care system [25].

In the current Mexican population, the use of health services was higher in women than in men, which agrees with previous studies. Colombian health service users (*n* = 11,121,054) were assessed in 2017 by means of clinical records and showed that women used health services more than men [23]. The assessment of 2244 German 19–79-year-old patients in 2020 reported that women visited doctors and used health prevention and promotion programs more often than men [24]. This fact may be due to various factors such as greater early identification of symptoms, biological differences, greater perception of the disease, and cultural aspects. Several authors have previously affirmed that women tend to make a greater search for preventive health services, which increases the probability of receiving diagnoses and treatments that require a higher use of health services [19,23,26].

In the current results, health services were most used by people aged ≥ 60 years old. This finding agrees with Colombian people, showing that people over fifty years old represent 60–72% of the health service use [27]. This could be explained by the physiological changes that accompany their aging and by the higher prevalence of cardiovascular risk factors or chronic diseases such as hypertension, diabetes mellitus and MetS, which coincides with our study.

The current results of the association analysis show that people with a low level of studies are more likely to use health services. Previous studies showed a positive association between years of study and use of health services [18,19,23]. It has also been pointed out that a higher education of the individual and that of the parents allows for early identification of the symptoms of disease; so, it is expected that a highly educated family will use more health services, especially health preventive services [25]. However, in the current results, the group with the lowest educational level was associated with greater use of health services. This finding is similar to findings reported in other studies [19,28,29].

In the study carried out in 408 family nuclei and 1244 individuals from Cuba in 2013, they reported that the individuals who make the most use of services are those with the least education and consider that it could be more related to age (over 65 and under 10 years) than with schooling itself. In this sense, it was previously pointed out [19] that people of low social class and with less education were associated with greater use of health services. The greater use is mediated by a greater need in health. People with voluntary health insurance tend to use health services more. However, as this characteristic is associated with a higher income, it favors use only in the least needy groups, thus fueling inequity.

Scientific evidence showed that the population with cardiovascular risk factors and MetS and medical insurance had higher rates of use of health care services [18,19,23]; however, the current findings showed a low percentage of health service use (48%) among current Mexican insurers.

The use of health services to control cardiovascular risk factors and MetS is highly relevant, particularly for low- and middle-income countries due to the high prevalence of cardiovascular risk factors and MetS and the financial and social costs it represents, both for the health system and for patients and their families. For health service staff, it encourages having better organization and planning for cost-effective interventions, as well as the incorporation of multidisciplinary health teams in developing actions to prevent, promote, detect, diagnose, treat, and control cardiovascular risk factors and MetS [19,22,25]. For the eligible population, it would be convenient to have greater and better knowledge about MetS, diabetes mellitus, hypertension, and cardiovascular risk factors to better decide their own health. Low self-perception of risk, harmful habits, and low use of medical care has been associated with poor health outcomes, leading to a higher burden of disease [27,30].

## 5. Strengths and Limitations

The strength of this study is that, for the first time, the use of health services and its relationship with metabolic syndrome and the risk cardiovascular diseases has been described among Mexicans. This study highlights the need to design interventions directed at susceptible populations to reduce gaps in the use of health services among the population. In some analyzed variables (age range, educational level, region, diabetes mellitus, hypertension, and metabolic syndrome), there are differences between the results obtained in the crude analysis (percentages) and those obtained through the logistic regression model. The adjustment by other variables in the logistic regression model removed the confounding effects.

Limitations of the current study were the lack of information on what services were used, reasons for consultation, and the frequency of use of health services (number per person and month/year). It would have been desirable to know the exact number of consultations that users asked to stabilize the severity of their MetS. The possibility of using health services on a regular basis is an important indicator of access to health services, which is influenced by the provision of services and the perceived need by its users [19]. Another limitation is that the participants in the study were workers or pensioners of ISSSTE; participants would have more information and/or access to medical attention than the regular population if they worked in those hospitals. Lastly, there was a selection bias because the study was carried out in the ISSSTE hospitals, and the population affiliated with the ISSSTE who used the health services were mostly women; accordingly, that explained why there were more women than men in the sample size.

## 6. Conclusions

Among ISSSTE health service users, there is a positive, significant association between the use of health services and the presence of metabolic syndrome, obesity, diabetes, hypertension, hypertriglyceridemia, and hypercholesterolemia. Further studies are required in Mexico to study the determinants in the use and access to health services for chronic diseases and their risk factors, and these studies should be done on representative samples of all health services at the national level.

## Figures and Tables

**Table 1:** Use of health services according to sociodemographic characteristics.

Use of Health Services				
	Total (*n* = 4595)	No (*n* = 2204)	Yes (*n* = 2391)	*p* *
Sex				
Male (%)	33.2	38.3	28.5	
Female (%)	66.8	61.7	71.5	<0.001
Age				
20–39 years old (%)	32.2	38.8	25.9	
40–59 years old (%)	50.0	49.2	50.9	
≥60 years old (%)	17.8	12.0	23.2	<0.001
Region				
Central-West (%)	29.5	30.1	29.0	
Central (%)	25.5	25.1	25.8	
North (%)	22.8	24.4	21.3	
South-South East (%)	22.2	20.4	23.9	0.008
Scholar level				
Lower than primary school (%)	0.3	0.1	0.4	
Primary school (%)	6.8	4.8	8.7	
High school (%)	20.5	17.9	23.0	
Preparation for university (%)	26.9	26.7	27.0	
University (%)	45.5	50.5	40.9	<0.001
Frequency of health services use				
Always (%)	11.8	0	22.7	
Usually (%)	13.5	0	26.0	
Sometimes (%)	26.7	0	51.3	
Rarely (%)	33.8	70.4	0	
Never (%)	14.2	29.6	0	<0.001

* *p* values “yes” vs. “no” were obtained by χ^2^.

**Table 2:** Use of health services according to prevalence of metabolic syndrome and its components.

Use of Health Services				
	Total (*n* = 4595)	No (*n* = 2204)	Yes (*n* = 2391)	*p* *
Metabolic syndrome (MetS)				
With MetS (%)	56	61	51.3	
Without MetS (%)	44.0	39	48.7	<0.001
Fasting hyperglycaemia (≥100 mg/dL)				
No (%)	65.0	69.1	61.2	
Yes (%)	35.0	30.9	38.8	<0.001
Hypertriglyceridemia (≥150 mg/dL)				
No (%)	42.9	46.2	39.9	
Yes (%)	57.1	53.8	60.1	<0.001
Low HDL-cholesterol (<40 mg/dL men; <50 mg/dL women)				
No (%)	39.2	41.2	37.3	
Yes (%)	60.8	58.8	62.7	0.009
Abdominal obesity				
No (%)	52.0	54.7	49.5	
Yes (%)	48.0	45.3	50.5	<0.001
Hypertension (≥130/85 mg/dL)				
No (%)	52.4	55.7	49.4	
Yes (%)	47.7	44.3	50.6	<0.001

Abbreviations: With MetS, with metabolic syndrome; Without MetS, without metabolic syndrome. * *p* values “yes” vs. “no” were obtained by χ^2^.

**Table 3:** Use of health services according to cardiovascular risk factors.

Use of Health Services				
	Total (*n* = 4595)	No (*n* = 2204)	Yes (*n* = 2391)	*p* *
Smoking				
Smokers (%)	17.4	19.5	15.5	
Ex-smokers (%)	17.8	16.6	19.0	
Never smoked (%)	64.8	63.9	65.5	0.001
BMI				
Normal weight (%)	25.1	26.2	24.0	
Overweight (%)	43.2	43.6	42.8	
Obesity (%)	31.7	30.2	33.2	0.053
Diabetes				
No (%)	80.3	84.7	76.2	
Yes (%)	19.7	15.3	23.8	<0.001
Hypertension				
No (%)	52.4	55.7	49.4	
Yes (%)	47.6	44.3	50.6	<0.001
Hypertriglyceridemia				
No (%)	42.9	46.2	39.9	
Yes (%)	57.1	53.8	60.1	<0.001
Low HDL-cholesterol (<40 mg/dl men; <50 mg/dl women)				
No (%)	39.2	41.2	37.3	
Yes (%)	60.8	58.8	62.7	0.009
High total cholesterol (≥200 mg/dl or treated)				
No (%)	37.2	39.7	34.9	
Yes (%)	62.8	60.3	65.1	0.001

Abbreviations: BMI, body mass index. * *p* values “yes” vs. “no” were obtained by χ^2^.

**Table 4:** Multivariate model of logistic regression to estimate the probability of use of health services according to sociodemographic characteristics, cardiovascular risk factors and metabolic syndrome in Mexican adults (unadjusted OR).

Use of Health Services		
	OR * (95%CI)	*p*
**Sociodemographic Characteristics**		
Sex		
Female	1.56 (1.37–1.76)	<0.001
Male	1.00 (ref.)	
Age		
20–39 years old	0.35 (0.29–0.41)	<0.001
40–59 years old	0.54 (0.45–0.63)	<0.001
≥60 years old	1.00 (ref.)	
Scholar level		
Lower than primary school	3.44 (0.92–12.72)	0.050
Primary school	2.23 (0.73–2.88)	<0.001
High school	1.59 (1.36–1.86)	<0.001
Preparation for university	1.25 (1.09–1.45)	0.002
University	1.00 (ref.)	
Region		
West	0.82 (0.70–0.97)	0.020
Central	0.88 (0.74–1.04)	0.124
North	0.74 (0.62–0.89)	0.001
South-South East	1.00 (ref.)	
Cardiovascular risk factors		
BMI		
Normal weight	0.80 (0.71–0.97)	0.019
Overweight	0.89 (0.77–1.01)	0.090
Obesity	1.00 (ref.)	
Smoking		
Smokers	0.78 (0.67–0.91)	0.002
Ex-smokers	1.11 (0.95–1.30)	0.177
Never smoked	1.00 (ref.)	
Diabetes		
Yes	1.73 (1.49–2.00)	<0.001
No	1.00 (ref.)	
Hypertension		
Yes	1.29 (1.14–1.45)	<0.001
No	1.00 (ref.)	
Hypertriglyceridemia		
Yes	1.30 (1.15–1.46)	<0.001
No	1.00 (ref.)	
Low HDL-cholesterol (<40 mg/dl men; <50 mg/dl women)		
Yes	1.17 (1.04–1.32)	0.009
No	1.00 (ref.)	
High total cholesterol (≥200 mg/dl or treated)		
Yes	1.23 (1.09–1.39)	0.001
No	1.00 (ref.)	
Metabolic syndrome		
Metabolic syndrome		
Yes	1.48 (1.31–1.67)	<0.001
No	1.00 (ref.)	

* OR adjusted odds ratio.

**Table 5:** Multivariate model of logistic regression to estimate the probability of use of health services according to sociodemographic characteristics, cardiovascular risk factors and metabolic syndrome in Mexican adults (adjusted OR).

Use of Health Services		
	OR * (95%CI)	*p*
Sex		
Female	1.77 (1.54–2.03)	<0.001
Male	1.00 (ref.)	
Age		
20–39 years old	0.41 (0.34–0.51)	<0.001
40–59 years old	0.57 (0.47–0.69)	<0.001
≥60 years old	1.00 (ref.)	
Scholar level		
Lower than primary school	4.27 (0.90–20.24)	0.050
Primary school	1.52 (1.15–2.01)	0.003
High school	1.34 (1.13–1.59)	<0.001
Preparation for university	1.16 (0.99–1.35)	0.056
University	1.00 (ref.)	
Region		
West	0.76 (0.64–0.91)	0.002
Central	0.85 (0.71–1.02)	0.086
North	0.76 (0.63–0.91)	0.004
South-South East	1.00 (ref.)	
Diabetes		
Yes	1.37 (1.16–1.63)	<0.001
No	1.00 (ref.)	
Hypertriglyceridemia		
Yes	1.25 (1.09–1.43)	<0.001
No	1.00 (ref.)	

* OR adjusted odds ratio.

## Data Availability

There are restrictions on the availability of data for this trial, due to the signed consent agreements around data sharing, which only allow access to external researchers for studies following the project purposes. Requestors wishing to access the trial data used in this study can make a request to pep.tur@uib.es.
